# Effects of maternal ritodrine hydrochloride administration on the heart rate of preterm fetal sheep with intraamniotic inflammation

**DOI:** 10.1371/journal.pone.0265872

**Published:** 2022-03-31

**Authors:** Tsuyoshi Murata, Hyo Kyozuka, Shun Yasuda, Toma Fukuda, Teruyoshi Tanaka, Keiya Fujimori

**Affiliations:** 1 Department of Obstetrics and Gynecology, Fukushima Medical University School of Medicine, Fukushima, Japan; 2 Department of Biomolecular Science, Fukushima Medical University School of Medicine, Fukushima, Japan; AUSTRALIA

## Abstract

Ritodrine hydrochloride is used for pregnancy prolongation and intrauterine fetal resuscitation. However, its clinical significance in intraamniotic inflammation during preterm labor and intrauterine fetal distress is unclear. We investigated the effects of maternal ritodrine hydrochloride administration (MRA; 200 μg/min for 2 h, followed by 800 μg/min for 2 h after 24 h) on fetal physiological parameters. For this purpose, we used chronically instrumented pregnant sheep at 113–119 d (term = 145 d) of gestation without (Group 1, n = 5) and with (Group 2, n = 5) intraamniotic inflammation induced by lipopolysaccharide injection into the amniotic cavity. The changes in fetal heart rate (FHR) and short-term variability (STV) and long-term variability (LTV) in FHR, fetal blood pressure, and fetal arterial blood gas (FABG) values were measured before and at 1 and 2 h after initiating MRA. Before MRA, all parameters were similar between Groups 1 and 2; however, there was significantly higher STV in Group 2 than in Group 1 before MRA at 800 μg/min, significantly higher partial arterial pressure of carbon dioxide in FABG in Group 2 than in Group 1 before MRA at 200 μg/min, and significantly lower blood glucose (BG) in Group 2 than in Group 1 before MRA at 800 μg/min. One hour after MRA, the FHR, STV, and LTV were significantly higher at 800 μg/min than those at the baseline in Group 1, as determined by the Friedman test; however, no significant difference was observed in Group 2. Additionally, the FABG pH significantly decreased 1 h after MRA at 800 μg/min in Group 2, whereas FABG lactate and BG significantly increased 2 h after MRA at 800 μg/min in Groups 1 and 2. Thus, short-term MRA at 800 μg/min increased the FHR, STV, and LTV significantly; these values were further modified under intraamniotic inflammation.

## Introduction

Ritodrine hydrochloride has been used as a beta-sympathomimetic agent for pregnant women with preterm labor (PTL) or intrauterine fetal distress. It predominantly interacts with the beta-2 receptors located in the uterus, suppressing undesirable uterine contractions [1]. Studies have shown that maternal ritodrine hydrochloride administration (MRA) prolongs pregnancies by at least 48 h, enabling the completion of antenatal corticosteroid therapy, thereby promoting the maturation of fetal lungs and allowing the transfer of pregnant women to a perinatal center [2–4]. Moreover, MRA, for acute tocolysis in patients experiencing excessive uterine contractions during labor, rapidly eliminates uterine contractions and improves the blood flow between the uterus and placenta to facilitate intrauterine fetal resuscitation in term and preterm gestations [5–8]. Furthermore, MRA is often performed under intraamniotic inflammatory conditions, which frequently accompany PTL and intrauterine fetal distress [9–12].

Long-term MRA is not recommended because it elicits serious maternal adverse effects, such as pulmonary edema, granulocytopenia, and rhabdomyolysis [13–15]. In 2013, the US Food and Drug Administration and European Medicines Agency recommended against the long-term use (over 48 h) of beta-sympathomimetic agents in pregnant women [16, 17]. Meanwhile, Japanese obstetricians are likely to select ritodrine hydrochloride as first-line therapy for tocolysis in patients with PTL [13, 18]. This is because the guidelines for obstetric practice in Japan have not renounced MRA over 48 h [18–21], even in the latest version published in 2020, whereas short-term MRA is recommended for tocolysis. However, evidence for the beneficial effects of even short-term MRA is still lacking [22], partially because there are few clarifications regarding the effects of short-term MRA on fetal conditions. Although previous studies have shown that MRA causes fetal tachycardia [23–25], its clinical significance is still unclear. Similarly, although recent studies have reported that MRA is associated with increased incidence of childhood asthma and wheezing in offspring [26, 27], it is unclear whether short-term MRA exhibits the same relationship. Furthermore, the effects of MRA on fetuses exposed to intraamniotic inflammation have not been reported.

The variability in fetal heart rate (FHR) reflects fetal autonomic functions and, in turn, fetal conditions [28, 29]. Although FHR variability could be affected by tocolytic agents, there is limited evidence regarding the effects of MRA on FHR variability, that is, MRA has been associated with decreased FHR variability under limited conditions [30, 31]. Therefore, we investigated the physiological changes in fetuses, including FHR variabilities, caused by MRA with and without intraamniotic inflammation using an appropriate animal model. Given that the fetal sheep weight is concordant with human fetal weight and that autonomic functions and reactions are similar to those in humans, sheep are suitable animals for the analysis of fetal physiology [32–35]. Thus, we used chronically instrumented pregnant sheep as the animal model.

There is a need to clarify the effects of short-term MRA on fetal physiological parameters with and without intraamniotic inflammation for the safe clinical implementation of short-term MRA. We hypothesized that, after administering a beta-sympathomimetic agent, the fetus would experience tachycardia and a decrease in FHR variability via autonomic responses, regardless of the presence of intraamniotic inflammation. To validate this hypothesis, we investigated the effects of short-term MRA on chronically instrumented fetal sheep by analyzing fetal physiological parameters under physiological and intraamniotic inflammatory conditions—the latter induced by lipopolysaccharide (LPS) administration into the amniotic fluid cavity.

## Materials and methods

### Chronically instrumented pregnant sheep model

This study was approved by the Animal Ethics Committee of Fukushima Medical University (No.: 28042: for 2016–2018, 30108: for 2018–2020, 2020058: for 2020–2021, and 2021017: for 2021–2023); we followed the guidelines for the care and use of animals mandated by the local institution. The researchers who treated pregnant sheep models complied with the ethical guidelines and regularly attended lectures on animal care and ethics of animal experimentation. The authors of this paper complied with ARRIVE guidelines when reporting this study.

Ten pregnant Corriedale/Suffolk sheep were purchased from a breeder (Japan-lamb Co., Ltd., Fukuyama City, Japan) and transferred to our animal laboratory facility via a truck more than five days prior to surgery. Two sheep were transferred at a time, maintained in individual metabolic cages in the same room at 18°C ± 2°C, and subjected to 12-h light/dark cycles (07:00–19:00 light; 19:00–07:00 dark) with controlled access to the food recommended for sheep breeding (alfalfa hay, 2.0 kg/day) and free access to the mineral block and water. The sheep were surgically instrumented between 113 and 119 d of gestation (term = 145 d). The sheep were not allowed access to food and water from one day before surgery until after the surgery.

On the day of surgery, general anesthesia was induced from 07:00 by intramuscular administration of 0.2 mg/kg xylazine (Bayer Yakuhin Ltd., Osaka City, Japan). Subsequently, 3 mg/kg/min dexmedetomidine hydrochloride (Maruishi Pharmaceutical Co., Ltd., Osaka City, Japan) [36] was administered via a peripheral intravenous line on the front right leg of the sheep obtained after xylazine administration. Acetaminophen (Terumo Co., Ltd., Tokyo, Japan) (1,000 mg/body/30 min) and antibiotics (1 g flomoxef sodium/body; biological half-life: 49.2 min; Shionogi Co., Ltd., Tokyo, Japan) were intravenously administered before surgery to control surgical pain and surgical site infection, respectively. During anesthesia, 100% oxygen was administered via a mask suitable for a sheep’s face. A researcher handled the infusion of anesthesia and observed the respiratory conditions and surgical pain levels of the sheep throughout the procedure. In the supine position, surgical sites were prepared by shearing the wool and using standard sterile procedures.

Using an aseptic technique, a midline incision was made on the abdominal skin. The fetal head was delivered through a partial hysterotomy and covered with a surgical glove filled with warm saline to prevent breathing. Polyvinyl catheters (1.2 and 1.8 mm inner and outer diameters, respectively; Imamura Co., Ltd., Tokyo, Japan) were placed into the fetal carotid arteries, jugular veins, and amniotic cavities. Catheters were placed into the fetal carotid arteries for blood collection and fetal blood pressure (FBP) measurement, fetal jugular veins for drug injection, and amniotic cavities for the administration of drugs. Electrodes attached to polyvinyl-coated stainless-steel wires (Tesco Co., Ltd., Tokyo, Japan) were placed on the trunks of the fetuses and to the bilateral upper limbs to record the fetal electrocardiograms. Thereafter, the fetuses were returned into the uterus, and the uterus and the incision on the skin were sutured using an absorbable suture. Furthermore, polyvinyl catheters were placed into the maternal femoral arteries for maternal arterial blood pressure measurement and veins for drug injection. All fetal catheters and electrodes and maternal catheters were exteriorized through the flank of the maternal sheep.

After the procedures, anesthesia was ceased, the intravenous catheter inserted in the front right leg was removed, and the animals were housed again in individual metabolic cages. All catheters inserted in the fetal and maternal arteries were maintained by continuously infusing heparin solubilized in physiological saline solution at 20 IU/mL at a rate of 0.5 mL/h. The catheter inserted in the maternal femoral vein was used to administer crystalloid fluid to maintain the body fluid balance, as well as the following drugs after surgery: acetaminophen (1,000 mg/body/30 min) was intravenously administered every 8 h to control postoperative pain and antibiotics were intravenously administered (1 g flomoxef sodium/body) every 12 h to prevent infections at the site of incision until the experiments began. All sheep quickly recovered from the anesthesia and exhibited a healthy appearance, a good appetite, and no difficulty standing when observed 2 h after anesthesia, thereby showing no signs of uncontrolled pain. We defined D0 as the date of the experiment on which maternal and fetal conditions, such as FHR, FHR variability, FBP, and fetal arterial blood gas (FABG) values, were confirmed to be stable; it was set as the baseline to secure the stable measurement of physiological parameters. The D0 dates ranged from days 0 to 4 post-surgery. Animal health and behavior were carefully observed at least every 6 h during the course of the experiment.

### Experimental protocol (Fig 1)

**Fig 1:**
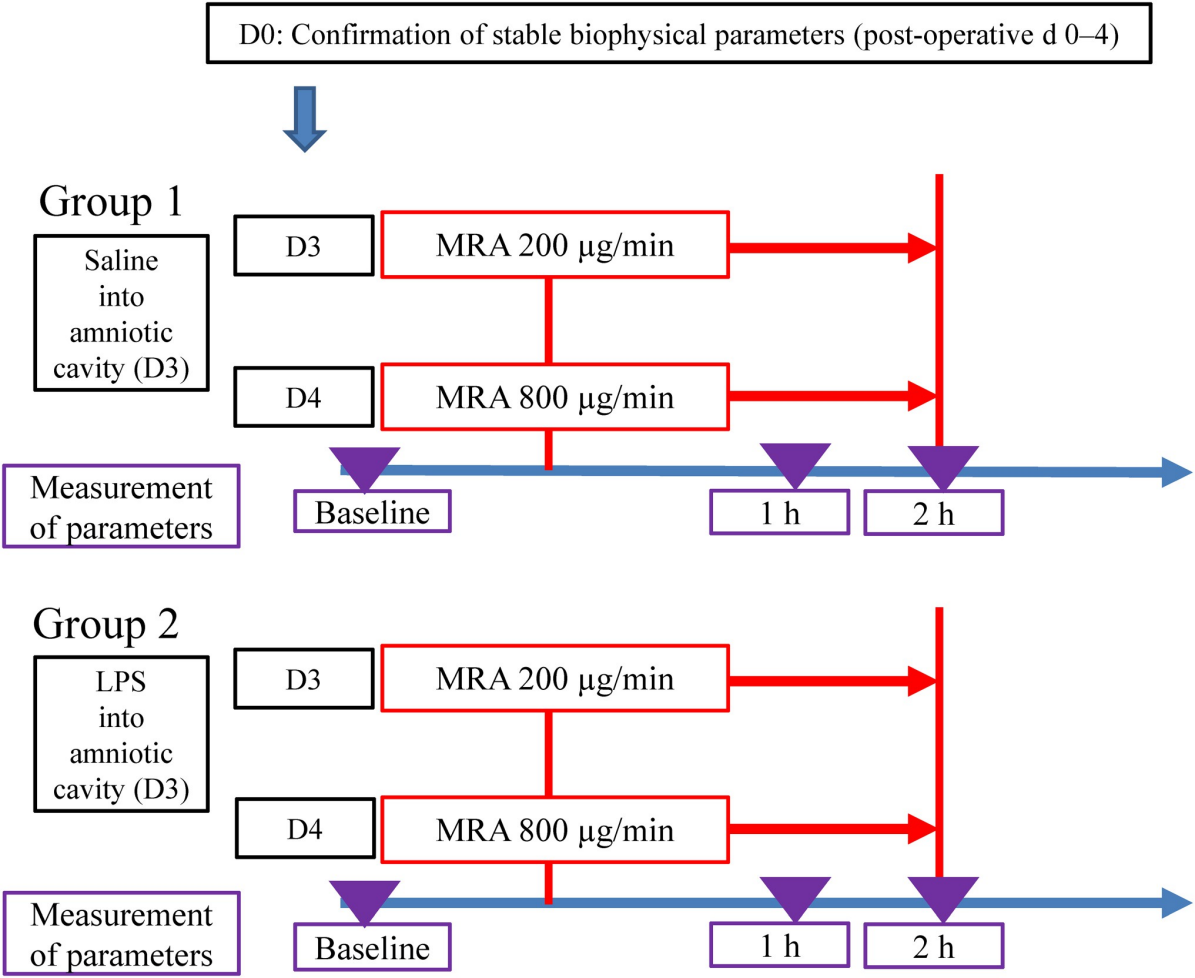
Experimental protocol. LPS: lipopolysaccharide, MRA: maternal ritodrine hydrochloride administration.

We randomly divided the sheep into the following groups: Group 1 (physiological group, n = 5) and Group 2 (intraamniotic inflammation group, n = 5). This randomization was made at the time of sheep allocation by the breeder to the first and second cage; we did not decide which sheep entered the first or second cage. The sheep entering the first cage were assigned to Group 1. The demographic characteristics of maternal sheep in both groups are summarized in Table 1. Information about maternal age and weight were lacking. No sheep in this study had any comorbidities during the experiment.

**Table 1:** Comparison of the demographic characteristics of maternal sheep between Groups 1 and 2.

	Gestational age at the time of surgery (days, median)	Recurrent cesarean section (n)	Singleton pregnancies (n)	Postoperative day at D0 (days, median)	Fetal weight (g, median)
**Group 1 (n = 5)**	119.0 (113.5–119.0)	3	3	1.0 (0.0–1.0)	2940 (2865–3050)
**Group 2 (n = 5)**	116.0 (113.5–118.0)	3	3	2.0 (1.0–4.0)	2900 (2800–3190)

Results are expressed as median (quartile range).

In Group 2, 50 μg of granulocyte-colony stimulating factor (G-CSF) (Neutrogin R. Chugai Co., Ltd., Tokyo, Japan) solubilized in 2 mL of physiological saline solution was administered daily via intravenous injection to the fetuses on D1 to D5. Furthermore, 50 mL of saline solution containing 40 mg of LPS (*Escherichia coli* 055:B5 endotoxin; Sigma-Aldrich Co., LLC., St. Louis, MO, USA) was administered by bolus infusion into the amniotic cavity on D3. The administration of G-CSF and LPS was based on the intraamniotic inflammation model described by Kyozuka et al. [32] and Watanabe et al. [37], which confirmed intraamniotic inflammation by histopathologic examinations after delivery. In Group 1 (as a control to Group 2), 2 mL of physiological saline solution was administered daily via intravenous injection into the fetuses on D1 to D5, and 50 mL of physiological saline solution was administered via bolus infusion into the amniotic cavity on D3.

In both groups, 40 mL of physiological saline solution containing ritodrine hydrochloride, with doses of 200 and 800 μg/min (D3 and D4, respectively), was injected for 2 h (20 mL/h) into the vein of each maternal sheep. The 24-h interval between drug injections was based on the half-life of ritodrine hydrochloride; it was also selected to minimize the differences in the phase of intraamniotic inflammation. The MRA doses of 200 and 800 μg/min have been validated for use in short-term tocolysis to treat PTL and for use in acute tocolysis to treat intrauterine fetal distress, respectively [5, 6, 13, 38]. As short-term MRA is recommended in clinical settings, and the duration of MRA to treat intrauterine fetal distress is clinically up to 2 hours, we comprehensively examined the effect of MRA for 2 h. The concentration of ritodrine hydrochloride in fetal serum was analyzed by liquid chromatography with tandem mass spectrometry (LC-MS/MS) [39] to confirm that fetal physiological changes were caused by MRA. The experimental procedure was started within 24 h of physiological saline or LPS administration into the amniotic cavity because acute and subacute intraamniotic inflammation frequently accompany PTL and intrauterine fetal distress [9–12]; a single dose of 40 mg of LPS can lead to a high degree of inflammation and rapid intrauterine fetal death (IUFD) after the administration period [32]. The concentration of interleukin-6 (IL-6) in fetal serum was analyzed by sandwich enzyme-linked immunosorbent assay (ELISA) [11, 40–42] to confirm fetal inflammatory responses at the time of the experiment.

FHR, FHR short-term variability (STV), FHR long-term variability (LTV), FBP as systolic blood pressure (SBP) and diastolic blood pressure (DBP), and FABG (including the pH, partial arterial pressure of carbon dioxide [PaCO_2_], partial arterial pressure of oxygen [PaO_2_], base excess [BE], lactate, and blood glucose [BG]) were measured at three time points: at baseline (before MRA) and 1 and 2 h after initiating MRA. Sampling for the measurement of these parameters was performed at specific time points to ensure stable values without artifacts of each parameter. The sheep number was not disclosed to the researcher when each parameter was being measured so that the researcher could not recognize which group of sheep was being handled.

After the experiment, acetaminophen (1,000 mg/body/30 min) was considered to be intravenously administered to control post-experimental pain, if needed; however, no sheep showed any signs of post-experimental pain. Maternal and fetal catheters were quickly removed after IUFD. Fetuses were vaginally delivered as stillbirths without any catheters. Healthy maternal sheep that survived were returned to the breeder.

A humane endpoint was used to recognize early markers associated with death, poor prognosis, deteriorated quality of life, or specific signs of severe suffering or distress, and identify the point at which the animals needed to be euthanized. Researchers carefully evaluated the humane endpoint based on the anticipated clinical, physiological, and behavioral signs. These included unhealthy appearance, loss of appetite, abnormal behavior, pathological changes, reduced mobility, or abnormal body posture. Once a decision for euthanasia was made, the procedure was performed as soon as possible, generally within half a day. Animals were euthanized by intravenously administering thiopental and potassium chloride. One sheep in Group 1 was euthanized, as it met the endpoint criteria of unhealthy appearance, loss of appetite, and difficulty standing after undergoing the experimental protocol. No sheep died before meeting the criteria for euthanasia.

### Measurement of FHR

FHR was measured using an acquisition system (Power Lab; AD Instruments, Sydney, Australia). To calculate the FHR, individual R waves from the fetal electrocardiograms with a sampling rate of 1 kHz were sequentially identified. The distance between consecutive peaks of R waves was measured, converted to beats/min, and then expressed as a line chart. Twenty minutes of stable baseline FHR recording was converted to an hourly value by counting 20 points in the line charts.

### Quantification of variability

FHR variability was quantified by transferring the electrocardiographic signals, acquired during FHR measurements with the Power Lab system, and the ATM 1308 Variability Calculate System (Atom Medical Tokyo, Japan), an online animal experimental data display/recording device. The method of calculating FHR variability has been previously described [31, 32, 43, 44]; the resting rate (RR) interval of the acquired FHR signals was converted into beats/min and the difference in the RR interval between the contiguous intervals was calculated. STV and LTV were denoted when the directions of interval FHR were different and the same for two contiguous intervals, respectively. These values were accumulated for 100 consecutive RR intervals and then reset. STV and LTV were measured at a stable segment of a 20-min FHR, that is, using the same segment as the FHR measurement, at each time point and were each defined as hourly values.

### Measurement of FBP

FBP was measured using the acquisition system (Power Lab). The SBP and DBP were evaluated as FBP for each fetus. A stable 20-min FBP was selected and was converted to an hourly value by counting 20 points.

### Analysis of FABG

Fetal arterial blood was collected using a heparinized 1-mL plastic syringe and analyzed for FABG (pH, PaCO_2_, PaO_2_, BE, lactate, and BG) using a Corning 170 pH/Blood Gas Analyzer system (AACC, Washington DC, USA).

### Measurement of ritodrine hydrochloride concentration in fetal serum

The ritodrine hydrochloride concentration in fetal serum was analyzed by LC-MS/MS [39]. Blood was obtained from the fetal artery. The serum sample was obtained after centrifugation at 3,000 rpm for 10 min and stored at -80°C until further analysis. The serum sample (20 μL) was pipetted into a microtube, and 80 μL of acetonitrile (0.1% formic acid) was added to precipitate the proteins. After vigorous mixing, the sample was centrifuged (10,000 rpm, 30 s). For sample clean-up, a monolithic silica spin column chemically bonded with TiO_2_ and ZrO_2_ (GL Sciences Inc., Tokyo, Japan) was used according to the manufacturer’s instructions. After sample clean-up, 1.0 × 10^−3^ μg of salbutamol was added as the internal standard. The concentration of ritodrine hydrochloride was analyzed by C18 reverse-phase LC coupled to high resolution-MS. An aliquot of the sample was loaded onto a high-performance liquid chromatography (HPLC)-MS/MS system (HPLC, UltiMate 3000; MS, TSQ Vantage, Thermo Scientific, Waltham, MA, USA) controlled using Xcalibur (v.2.2, Thermo Scientific). The concentration of ritodrine hydrochloride in each sample was determined from a calibration curve constructed by plotting the peak area ratio (the peak area of ritodrine hydrochloride to that of salbutamol).

### Measurement of IL-6 concentration in fetal serum

The IL-6 concentration in fetal serum was analyzed using an ovine-specific sandwich ELISA Kit for IL-6 (SEA079Ov; CLOUD-CLONE CORP., Houston, USA) [42], which includes a pre-coated, ready-to-use 96-well strip plate, IL-6 standards, detection reagents, TMB substrate, wash buffer, plate sealer, standard diluent, assay diluents, and stop solution. Blood was obtained from the fetal artery. The serum sample was obtained after centrifugation at 3,000 rpm for 10 min and stored at -80°C until further analysis. All reagents were stored according to information labels on the vials. The samples were then defrosted at room temperature (25°C). The reagents were prepared and assays were performed according to the manufacturer’s instructions. The optimal sample dilutions for analysis were determined by conducting preliminary experiments to determine the validity of the dilution; the final analyses were conducted with 2 or 10 dilutions. All samples and standards were assayed in duplicate. The concentration of IL-6 was measured at 450 nm using a spectro-photometer (Molecular Devices SpectraMax Plus384). The standard curve (a five-parameter logistic standard curve) was constructed using Molecular Devices SoftMax Pro ver.6. The original detection range of this kit was 7.8–500 pg/mL.

### Statistical analysis

First, we determined the differences in the baseline values of FHR, STV, LTV, FBP, and FABG between Groups 1 and 2 prior to MRA at doses of 200 μg/min and 800 μg/min using the Mann–Whitney *U* test. Second, we determined the differences in the FHR, STV, LTV, FBP, and FABG values in Groups 1 and 2 after MRA using the Friedman test. A post hoc analysis using the Bonferroni correction was performed when an overall significant difference was observed.

Additionally, we determined the differences in the ritodrine hydrochloride concentration in fetal serum at the baseline and at 1 h and 2 h after MRA between Groups 1 and 2, using the Mann–Whitney *U* test. We also determined the differences in the baseline values of the IL-6 concentration in fetal serum between Groups 1 and 2 before MRA at doses of 200 μg/min and 800 μg/min using the Mann–Whitney *U* test.

Based on the small sample size and data distribution determined from a histogram, the Shapiro–Wilk test, and skewness analysis, we performed non-parametric analyses. SPSS version 26 (IBM Corp., Armonk, NY, USA) was used for the statistical analyses. Results with *p*-values < 0.05 were considered statistically significant. Data are presented as median values and the quartile range.

## Results

### Analysis of baseline physiological parameters in Groups 1 and 2

The median FHR, STV, LTV, FBP, and FABG values at baseline in both groups are summarized in Table 2. There were no significant differences in the FHR, LTV, or FBP values between Groups 1 and 2. However, there was a significantly higher STV in Group 2 than in Group 1 before MRA at a dose of 800 μg/min (*p* = 0.032), significantly higher PaCO_2_ in Group 2 than in Group 1 before MRA at a dose of 200 μg/min (*p* = 0.032), and significantly lower BG in Group 2 than in Group 1 before MRA at a dose of 800 μg/min (*p* = 0.032).

**Table 2:** Comparison of baseline physiological parameters between Groups 1 and 2 using Mann–Whitney *U* test.

	G1 MRA 200	G2 MRA 200	p-value	G1 MRA 800	G2 MRA 800	*p*-value
**FHR (bpm)**	188.05 (177.10–216.18)	203.35 (170.65–218.05)	0.841	184.90 (165.30–187.48)	187.05 (184.33–196.33)	0.151
**STV (bpm/100)**	65.32 (61.05–99.80)	93.44 (78.00–125.70)	0.222	61.49 (55.92–86.53)	99.34 (84.88–110.39)	0.032*
**LTV (bpm/100)**	25.95 (16.01–55.09)	39.85 (28.87–76.55)	0.222	13.36 (9.57–50.66)	46.53 (28.49–62.70)	0.151
**SBP (mmHg)**	65.44 (51.80–77.67)	56.89 (50.02–64.22)	0.421	61.91 (53.95–84.22)	52.61 (50.46–55.20)	0.151
**DBP (mmHg)**	44.40 (34.97–59.07)	38.94 (35.55–45.61)	0.690	39.26 (34.28–70.85)	37.54 (36.10–39.97)	0.841
**FABG**						
**pH**	7.379 (7.363–7.395)	7.351 (7.323–7.395)	0.421	7.376 (7.348–7.385)	7.383 (7.348–7.432)	0.548
**PaCO**_**2**_ **(mmHg)**	41.60 (33.70–46.05)	53.80 (46.05–54.40)	0.032*	43.60 (42.90–48.40)	46.10 (44.80–49.65)	0.222
**PaO**_**2**_ **(mmHg)**	24.60 (19.30–27.35)	19.50 (18.20–21.40)	0.151	21.60 (18.60–24.55)	16.00 (15.80–24.05)	0.421
**BE (mmol/L)**	0.40 (-5.20–1.70)	2.20 (-0.50–5.35)	0.222	0.50 (-1.10–2.40)	1.70 (1.10–5.45)	0.222
**Lactate (mmol/L)**	1.50 (1.20–1.80)	2.10 (1.55–3.10)	0.095	1.60 (1.45–3.30)	2.10 (1.50–2.70)	0.841
**BG (mg/dL)**	17.00 (15.00–26.00)	14.00 (10.50–18.50)	0.151	20.00 (15.00–25.00)	12.00 (11.00–13.50)	0.032*

Results are expressed as median (quartile range). Results with *p*-values < 0.05 were considered statistically significant and indicated with *.

G1: Group 1, G2: Group 2, MRA 200: maternal ritodrine hydrochloride administration at a dose of 200 μg/min, MRA 800: maternal ritodrine hydrochloride administration at a dose of 800 μg/min, FHR: fetal heart rate, STV: short-term variability of FHR, LTV: long-term variability of FHR, SBP: systolic blood pressure, DBP: diastolic blood pressure, FABG: fetal arterial blood gas, BE: base excess, BG: blood glucose, bpm: beats per minute.

### Analysis of physiological parameters before and after MRA in Groups 1 and 2

The changes in the FHR, STV, LTV, FBP, and FABG values in Groups 1 and 2 before and after MRA are summarized in Table 3. The FHR, STV, and LTV were significantly different in Group 1 after MRA at a dose of 800 μg/min (*p* = 0.022, *p* = 0.007, and *p* = 0.007, respectively). Although the same trend was observed in Group 1 after MRA at a dose of 200 μg/min, the difference was not significant. The FHR, STV, and LTV were not significantly different in Group 2 after MRA at doses of 200 μg/min and 800 μg/min. Moreover, the FBP was not significantly changed after MRA in Groups 1 and 2. Additionally, the FABG pH was significantly different in Group 2 after MRA at a dose of 800 μg/min (*p* = 0.022), and FABG lactate and BG values were significantly different in Group 1 after MRA at a dose of 800 μg/min and in Group 2 after MRA at both doses (*p* = 0.015, *p* = 0.011, *p* = 0.019, *p* = 0.015, *p* = 0.008, and *p* = 0.008 respectively).

**Table 3:** Comparison of physiological parameters before and after MRA in Groups 1 and 2 using Friedman test.

	Baseline	1 h	2 h	*p*-value
**G1 MRA 200**				
FHR (bpm)	188.05 (177.10–216.18)	197.30 (187.88–223.50)	212.70 (196.70–217.70)	0.076
STV (bpm/100)	65.32 (61.05–99.80)	79.87 (74.01–88.99)	104.21 (72.19–158.66)	0.247
LTV (bpm/100)	25.95 (16.01–55.09)	26.71 (22.57–36.50)	38.73 (25.74–63.62)	0.549
SBP (mmHg)	65.44 (51.80–77.67)	63.51 (53.36–75.74)	59.20 (54.58–76.14)	0.819
DBP (mmHg)	44.40 (34.97–59.07)	41.56 (34.86–59.67)	36.36 (35.74–59.53)	0.819
FABG				
pH	7.379 (7.363–7.395)	7.391 (7.357–7.400)	7.375 (7.341–7.405)	0.819
PaCO_2_ (mmHg)	41.60 (33.70–46.05)	41.20 (37.80–44.05)	40.20 (32.30–45.00)	0.449
PaO_2_ (mmHg)	24.60 (19.30–27.35)	21.20 (20.55–22.75)	21.00 (18.35–25.90)	0.678
BE (mmol/L)	0.40 (-5.20–1.70)	-1.70 (-2.55–1.65)	-0.40 (-7.70–2.40)	0.819
Lactate (mmol/L)	1.50 (1.20–1.80)	1.60 (1.25–2.05)	1.50 (1.15–2.15)	0.589
BG (mg/dL)	17.00 (15.00–26.00)	20.00 (16.50–25.00)	19.00 (16.00–31.50)	0.348
**G1 MRA 800**				
FHR (bpm)	184.90 (165.30–187.48)	230.25 (212.38–237.25)	222.05 (197.20–231.18)	0.022*
STV (bpm/100)	61.49 (55.92–86.53)	193.37 (126.52–293.60)	130.61 (93.30–190.61)	0.007*
LTV (bpm/100)	13.36 (9.57–50.66)	97.87 (62.93–137.57)	66.75 (43.46–98.76)	0.007*
SBP (mmHg)	61.91 (53.95–84.22)	64.48 (56.53–90.75)	65.20 (56.72–89.41)	0.247
DBP (mmHg)	39.26 (34.28–70.85)	37.04 (34.63–72.39)	37.86 (34.99–72.37)	0.165
FABG				
pH	7.376 (7.348–7.385)	7.379 (7.346–7.399)	7.388 (7.365–7.401)	0.165
PaCO_2_ (mmHg)	43.60 (42.90–48.40)	44.30 (41.40–45.05)	43.70 (41.10–45.00)	0.549
PaO_2_ (mmHg)	21.60 (18.60–24.55)	20.50 (16.75–24.75)	20.80 (17.05–23.05)	0.247
BE (mmol/L)	0.50 (-1.10–2.40)	0.90 (-2.25–2.10)	1.10 (-1.05–2.15)	0.549
Lactate (mmol/L)	1.60 (1.45–3.30)	2.70 (2.05–4.05)	2.90 (2.20–4.50)	0.015*
BG (mg/dL)	20.00 (15.00–25.00)	31.00 (17.50–43.00)	31.00 (23.00–51.50)	0.011*
**G2 MRA 200**				
FHR (bpm)	203.35 (170.65–218.05)	205.05 (178.35–210.48)	195.40 (174.63–199.73)	0.247
STV (bpm/100)	93.44 (78.00–125.70)	123.24 (68.99–195.51)	111.28 (59.11–120.71)	0.449
LTV (bpm/100)	39.85 (28.87–76.55)	85.84 (36.18–104.25)	34.50 (29.43–62.98)	0.247
SBP (mmHg)	56.89 (50.02–64.22)	57.66 (49.64–61.75)	59.85 (53.02–60.27)	0.247
DBP (mmHg)	38.94 (35.55–45.61)	36.53 (35.04–44.17)	37.91 (37.01–41.97)	0.449
FABG				
pH	7.351 (7.323–7.395)	7.368 (7.332–7.399)	7.348 (7.334–7.401)	0.692
PaCO_2_ (mmHg)	53.80 (46.05–54.40)	50.80 (45.85–52.10)	50.00 (47.05–51.75)	0.247
PaO_2_ (mmHg)	19.50 (18.20–21.40)	18.70 (16.65–23.70)	17.90 (12.30–19.65)	0.091
BE (mmol/L)	2.20 (-0.05–5.35)	0.80 (-0.45–6.50)	1.20 (-0.05–5.60)	0.819
Lactate (mmol/L)	2.10 (1.55–3.10)	2.80 (1.90–3.90)	2.70 (2.00–4.05)	0.019*
BG (mg/dL)	14.00 (10.50–18.50)	21.00 (17.50–33.00)	23.00 (20.50–34.00)	0.015*
**G2 MRA 800**				
FHR (bpm)	187.05 (184.33–196.33)	188.90 (183.98–193.65)	192.55 (184.28–196.15)	0.549
STV (bpm/100)	99.34 (84.88–110.39)	120.36 (83.33–232.62)	131.66 (68.75–145.87)	0.247
LTV (bpm/100)	46.53 (28.49–62.70)	86.76 (32.62–128.00)	43.55 (35.42–83.94)	0.449
SBP (mmHg)	52.61 (50.46–55.20)	52.20 (49.72–52.58)	51.16 (49.20–51.84)	0.165
DBP (mmHg)	37.54 (36.10–39.97)	35.56 (35.07–37.26)	34.39 (34.13–36.87)	0.074
FABG				
pH	7.383 (7.348–7.432)	7.348 (7.326–7.403)	7.346 (7.314–7.419)	0.022*
PaCO_2_ (mmHg)	46.10 (44.80–49.65)	49.50 (45.60–50.75)	46.40 (43.90–50.20)	0.247
PaO_2_ (mmHg)	16.00 (15.80–24.05)	16.30 (15.75–18.30)	16.40 (15.75–19.10)	0.692
BE (mmol/L)	1.70 (1.10–5.45)	2.00 (-1.05–3.75)	0.50 (-1.75–4.15)	0.074
Lactate (mmol/L)	2.10 (1.50–2.70)	3.50 (2.10–3.60)	3.50 (2.60–4.05)	0.008*
BG (mg/dL)	12.00 (11.00–13.50)	20.00 (12.50–25.00)	22.00 (18.50–31.50)	0.008*

Results are expressed as the median (quartile range). Results with *p*-values < 0.05 were considered statistically significant and indicated with *.

G1: Group 1, G2: Group 2, MRA 200: maternal ritodrine hydrochloride administration at a dose of 200 μg/min, MRA 800: maternal ritodrine hydrochloride administration at a dose of 800 μg/min, FHR: fetal heart rate, STV: short-term variability of FHR, LTV: long-term variability of FHR, SBP: systolic blood pressure, DBP: diastolic blood pressure, FABG: fetal arterial blood gas, BE: base excess, BG: blood glucose, bpm: beats per minute.

### Post hoc analysis of physiological parameters before and after MRA

We conducted post hoc analysis of changes in the FHR, STV, and LTV values in Group 1 after MRA at a dose of 800 μg/min, FABG pH in Group 2 after MRA at a dose of 800 μg/min, and FABG lactate and BG values in Group 1 after MRA at a dose of 800 μg/min and in Group 2 after MRA at both doses using the Bonferroni correction based on the results of the Friedman test. The results are summarized in Table 4. The FHR increased 1 h after MRA at a dose of 800 μg/min (*p* = 0.034) in Group 1, compared with the baseline value. The STV and LTV significantly increased 1 h after MRA at a dose of 800 μg/min in Group 1, compared with the baseline values (*p* = 0.005 and *p* = 0.005, respectively). The FABG pH values decreased 1 h after MRA at a dose of 800 μg/min (*p* = 0.034) in Group 2, compared with the baseline value; however, the FABG pH values were not within the range of fetal acidosis. The FABG lactate values increased 2 h after MRA at a dose of 800 μg/min in Groups 1 and 2 (*p* = 0.013 and *p* = 0.008, respectively). The FABG BG values increased 2 h after MRA at a dose of 800 μg/min in Groups 1 and 2 h after MRA at doses of 200 and 800 μg/min in Group 2 (*p* = 0.013, *p* = 0.013, and *p* = 0.008, respectively).

**Table 4:** Post hoc analysis of Friedman test results of physiological parameters before and after MRA using Bonferroni correction.

	Baseline		1 h	*p*-value	2 h	*p*-value
**G1 MRA800**						
FHR (bpm)	184.90 (165.30–187.48)	Ref	230.25 (212.38–237.25)	0.034*	222.05 (197.20–231.18)	0.081
STV (bpm/100)	61.49 (55.92–86.53)	Ref	193.37 (126.52–293.60)	0.005*	130.61 (93.30–190.61)	0.342
LTV (bpm/100)	13.36 (9.57–50.66)	Ref	97.87 (62.93–137.57)	0.005*	66.75 (43.46–98.76)	0.342
Lactate in FABG (mmol/L)	1.60 (1.45–3.30)	Ref	2.70 (2.05–4.05)	0.173	2.90 (2.20–4.50)	0.013*
BG in FABG (mg/dL)	20.00 (15.00–25.00)	Ref	31.00 (17.50–43.00)	0.464	31.00 (23.00–51.50)	0.013*
**G2 MRA200**						
Lactate in FABG (mmol/L)	2.10 (1.55–3.10)	Ref	2.80 (1.90–3.90)	0.053	2.70 (2.00–4.05)	0.053
BG in FABG (mg/dL)	14.00 (10.50–18.50)	Ref	21.00 (17.50–33.00)	0.173	23.00 (20.50–34.00)	0.013*
**G2 MRA800**						
pH in FABG	7.383 (7.348–7.432)	Ref	7.348 (7.326–7.403)	0.034*	7.346 (7.314–7.419)	0.081
Lactate in FABG (mmol/L)	2.10 (1.50–2.70)	Ref	3.50 (2.10–3.60)	0.246	3.50 (2.60–4.05)	0.008*
BG in FABG (mg/dL)	12.00 (11.00–13.50)	Ref	20.00 (12.50–25.00)	0.618	22.00 (18.50–31.50)	0.008*

Results are expressed as median (quartile range). Results with *p*-values < 0.05 were considered statistically significant and indicated with *.

G1: Group 1, G2: Group 2, MRA 200: maternal ritodrine hydrochloride administration at a dose of 200 μg/min, MRA 800: maternal ritodrine hydrochloride administration at a dose of 800 μg/min, FHR: fetal heart rate, STV: short-term variability of FHR, LTV: long-term variability of FHR, FABG: fetal arterial blood gas, BG: blood glucose, bpm: beats per minute.

### Analysis of ritodrine hydrochloride concentration in fetal serum before and after MRA in Groups 1 and 2

The ritodrine hydrochloride concentrations in fetal serum are summarized in Table 5. After MRA, ritodrine hydrochloride concentrations in Groups 1 and 2 increased from those at the baseline; however, there was no significant difference in the degree of increase between Groups 1 and 2 after MRA at doses of 200 μg/min and 800 μg/min. At the baseline in Group 2, ritodrine hydrochloride was detectable in extremely low levels; this was judged to be a measurement error and did not affect the study results.

**Table 5:** Comparison of ritodrine hydrochloride concentration in fetal serum before and after MRA between Groups 1 and 2 using the Mann–Whitney *U* test.

	Baseline	*p*-value	1 h	*p*-value	2 h	*p*-value
**G1 MRA200 (ng/mL)**	0.00 (0.00–0.00)	0.690	2.40 (1.80–3.60)	0.421	4.50 (2.45–4.85)	0.421
**G2 MRA200 (ng/mL)**	0.00 (0.00–0.05)		1.90 (1.15–3.45)		2.60 (2.05–4.15)	
**G1 MRA800 (ng/mL)**	0.00 (0.00–0.00)	0.690	4.40 (1.50–7.85)	0.841	6.00 (3.65–9.20)	1.000
**G2 MRA800 (ng/mL)**	0.00 (0.00–0.05)		4.30 (2.50–8.05)		4.90 (3.35–18.15)	

Results are expressed as the median (quartile range). Results with *p*-values < 0.05 were considered statistically significant.

G1: Group 1, G2: Group 2, MRA 200: maternal ritodrine hydrochloride administration at a dose of 200 μg/min, MRA 800: maternal ritodrine hydrochloride administration at a dose of 800 μg/min.

### Analysis of baseline IL-6 concentration in fetal serum in Groups 1 and 2

The IL-6 concentrations in fetal serum are summarized in Table 6. IL-6 concentrations were significantly higher in Group 2 before MRA at doses of 200 μg/min and 800 μg/min. Additionally, IL-6 concentrations before MRA at a dose of 800 μg/min were higher than those before MRA at a dose of 200 μg/min.

**Table 6:** Comparison of baseline IL-6 concentration in fetal serum between Groups 1 and 2 using the Mann–Whitney *U* test.

	G1 MRA 200	G2 MRA 200	p-value	G1 MRA 800	G2 MRA 800	*p*-value
**IL-6 (pg/mL)**	13.14 (0.93–19.09)	195.96 (135.91–619.89)	0.008*	47.36 (18.36–64.12)	482.34 (268.38–973.92)	0.008*

Results are expressed as the median (quartile range). Results with *p*-values < 0.05 were considered statistically significant and indicated with *.

G1: Group 1, G2: Group 2, MRA 200: maternal ritodrine hydrochloride administration at a dose of 200 μg/min, MRA 800: maternal ritodrine hydrochloride administration at a dose of 800 μg/min, IL-6: interleukin-6.

## Discussion

### Main findings

This is the first study to investigate the changes in fetal physiological parameters after short-term MRA at different doses, which has been validated for pregnancy prolongation and intrauterine fetal resuscitation, under both physiological and intraamniotic inflammatory conditions in pregnant sheep. Short-term MRA at a dose of 800 μg/min increased the FHR, STV, and LTV under physiological conditions but not under intraamniotic inflammation conditions.

### Interpretations

Ritodrine hydrochloride is a beta-2 adrenergic agonist; it stimulates maternal and fetal beta-adrenergic receptors in the bronchial and vascular smooth muscle cells. Fetal physiological parameters, such as FHR, FHR variability, and FBP, are well controlled by the autonomic nervous system, which is beneficial for the fetal immune response [33]. In this study, as understood from appropriate animal models, MRA affected several physiological parameters. According to previous studies that conducted similar experiments to identify these physiological parameters, we determined that the optimum number of sheep for inclusion in the study was 10. Considering the three Rs of animal experiments, using more than 10 sheep might not have changed the results pertaining to FHR, STV, and LTV significantly; therefore, we believe that this number of animals was appropriate. Additionally, the gestational age (GA) of sheep in this study was approximately 0.8 of the term at which we evaluated the fetal physiological conditions during development of the fetal hypothalamo-pituitary-adrenal axis [45]; moreover, 0.8 GA showed the greatest rate of fetal neural and cardiovascular development [46, 47]. In this study, we did not consider the difference between singleton/twin pregnancies and fetal sex. This is because Frasch et al. reported no significant difference in the physiological responses between singleton pregnancies and twin pregnancies in sheep at 0.8 GA [45], and Bennet et al. and Galinsky et al. reported similar physiological responses between male and female fetal sheep [48, 49]. In this animal model, an increase in the ritodrine hydrochloride concentration in fetal serum by MRA was confirmed by LC-MS/MS. Moreover, MRA to pregnant women allowed ritodrine hydrochloride to cross the placenta, thereby increasing drug concentrations in the umbilical vein [50]; MRA for 2 h to a pregnant sheep could directly stimulate fetal myocardium via autonomic responses, which is similar to that reported in humans [25]. This result reinforced the conclusion that the observed changes in the fetal physiological parameters were caused by increased ritodrine hydrochloride concentrations in the fetal serum by MRA.

Our results are consistent with those of previous studies, which showed increased FHR by MRA to pregnant women for 130 min [51] and by ritodrine hydrochloride administration into fetal sheep [52]. Although the clinical significance of tachycardia in fetuses is unknown [25], our results implied that MRA at a high dose, 1 h after initiation, significantly induced fetal autonomic changes, that is, the balance of sympathomimetic and parasympathetic responses. Contrary to our hypothesis, the STV and LTV may have increased by the autonomic condition affected by ritodrine hydrochloride, as an increase in beta-adrenergic activity resulted in increased FHR variability in animal experiments with brief and acute hypoxia [53]. Research on the influence of tocolytic agents on FHR variability is limited [30], and there is no clear evidence regarding the association between MRA and FHR variability. One study reported decreased FHR variability after MRA than after atosiban administration prior to 30 weeks of gestation [54]. Another study by our research group revealed decreased STV in pregnant women with fetal distress who underwent emergency cesarean section after short-term MRA at a high dose for intrauterine resuscitation [31]. Conversely, in the absence of labor contractions or other obstetrical drugs in our model, the 0.8 GA of the fetal sheep elucidates the ‘pure’ responses of mature autonomic systems after MRA because the FHR variability is affected by several clinical factors, including the GA, uterine contractions, and drugs used in the clinical setting [30]. Therefore, our study may be the first prospective analysis to provide insights into the effects of short-term MRA at different dosages, validated for treatments for PTL and intrauterine fetal distress, on FHR variability under physiological conditions. In contrast, there was no significant change in the FBP by MRA, suggesting that MRA has no significant effects on the FBP, one of the key indicators for fetal condition as well as FHR [55]; SBP and DBP in fetuses may have different implications with regard to the association of FHR and oxygenation [55]. Because FBP in humans is unavailable [55], this finding is one of the strengths of animal experiments.

A single dose of 40 mg of LPS administered into the intraamniotic cavity and recurrent G-CSF administration through the fetal vein caused intraamniotic inflammation, as LPS was administered at four times the dose of previous studies [56, 57], twice that reported by Watanabe et al. [37], and at the same dose reported by Kyozuka et al. [32]; this further showed that the second dosage led to a perimortem period [32]. Therefore, we evaluated the elevated IL-6 concentration in fetal serum to confirm the fetal inflammatory responses within a short period after LPS administration. Elevated IL-6 concentration in fetal serum confirmed intraamniotic inflammation and fetal responses at the time of the experiment, compared to placental histopathologic analysis after delivery. During intraamniotic inflammation, higher IL-6 concentrations in fetal serum before MRA at a dose of 800 μg/min than before MRA at a dose of 200 μg/min were noted, and a significant difference in the baseline values of STV between Groups 1 and 2 was noted before MRA at a dose of 800 μg/min in the present model; baseline samples in Group 2 were collected within 24 h (MRA at a dose of 200 μg/min) and after 24 h (MRA at a dose of 800 μg/min) of LPS administration into the amniotic cavities to minimize the differences in the phase of intraamniotic inflammation. Similarly, fetal responses under intraamniotic inflammation differed in previous studies, according to the phase of inflammation and the method of inflammation induction [58, 59]. However, previous studies showed conflicting results of fetal physiological parameters, based on the onset of intraamniotic inflammation (acute and chronic phase). One study reported tachycardia with intraamniotic inflammation [60]; however, others showed no significant changes in the FHR [32, 61]. Moreover, STV decreased with chronic inflammation in some studies [58, 62] but increased under fetal deterioration due to intraamniotic inflammation [32]. Moreover, hypotension was observed 2 d after repeated endotoxin exposure in the presence of chronic inflammation with impaired cardiac output due to decreased peripheral vascular resistance [61]. Therefore, a careful interpretation of the changes in fetal physiological parameters is needed according to the phase of intraamniotic inflammation, as the present study only focused on acute and subacute phases of intraamniotic inflammation and did not include a control group.

Unexpectedly, MRA did not alter the FHR, STV, and LTV values compared with baseline values during acute and subacute intraamniotic inflammation, although there was a consistent increase in the ritodrine hydrochloride concentration in fetal serum in both Group 2 and Group 1 after MRA at a dose of 800 μg/min. Although the effects of MRA on the fetal autonomic responses during intraamniotic inflammation have not been reported, one speculation is that fetal autonomic responses disappear upon exposure to inflammatory mediators [63]. Frasch et al. reported that inducing sepsis in animals resulted in the loss of inhibitory activity by the cholinergic anti-inflammatory pathway with decreased FHR variability, thereby leading to decreased vagal modulation [64]. Additionally, our study also showed that fetal cardiovascular responses on FHR during fetal distress such as hypoxemia may be decreased due to the collapse of the autonomic responses [33]. Thus, during intraamniotic inflammation, fetal inflammatory responses, and fetal distress, the fetal sheep might have experienced decreased autonomic responses after MRA. Similarly, the effect of decreased FABG pH with intraamniotic inflammation on fetal autonomic responses has also been reported in other studies. For example, Tjen-A-Looi et al. reported that the withdrawal of sympathetic tone and increased parasympathetic activity affecting the cardiovascular responses were mediated by splanchnic nerves at a low pH, which was caused by hypercapnia in rats [65]. Although the reduction of FABG pH 1 h after MRA was small and FABG PaCO_2_ was not significantly changed in Group 2 after MRA, this study revealed significantly increased FABG lactate 2 h after MRA at a dose of 800 μg/min in both Group 1 and Group 2; thus, increased FABG lactate may reflect the potential acidotic condition of the fetuses rather than pH [66, 67]. Additionally, there was a significant increase in FABG BG 2 h after MRA at a dose of 800 μg/min in both Groups 1 and 2, although FABG BG was significantly lower in Group 2 at the baseline. These factors may have affected fetal autonomic conditions. However, as these changes both occurred 2 h after MRA, the association between increased FABG lactate and BG and unchanged physiological parameters both 1 h and 2 h after MRA in Group 2 remains to be studied. As there have been few reports into the relationship between fetal lactate and BG and FHR variability, the potential effects of fetal lactate and BG changes on the results of this study should be elucidated in further studies.

### Clinical and research implications

The specific findings of FHR patterns on intraamniotic inflammation have not yet been revealed in a clinical setting, and the early clinical detection of intraamniotic inflammation remains a challenge for obstetricians. Physicians should be aware that the interpretation of FHR monitoring during the course of intraamniotic inflammation can be misleading [68], and the effects of MRA on fetal autonomic responses may be modified depending on the phase of inflammation. However, these modified parameters, especially FHR variability, may appear rapidly, reflecting real-time autonomic responses and may help identify the existence of early-stage intraamniotic inflammation [69]. As the early identification of intraamniotic inflammation has been an unresolved problem, we speculate that short-term MRA could mimic a loading dose, which detects the change in autonomic responses in a fetus; therefore, it could be a potential clinical marker of intraamniotic inflammation.

### Strengths and limitations

The major strength of the present study was the acquisition of FHR and FHR variability on a beat-to-beat basis using electrodes, which is more reliable than external monitoring with Doppler, because of unstable signal accuracy in terms of timing. Based on this method, we analyzed not only LTV, which is a popular metric in clinical settings and is usually estimated by visual interpretation, but also STV, which is calculated using the difference in the time interval between consecutive beats measured using R-R wave period differences in the ECG [31, 32, 43, 44, 70]. Both STV and LTV were significantly increased by MRA under physiological conditions in the present study, reflecting fetal autonomic responses. Specifically, STV is associated with severe fetal acidosis and stillbirth and is considered a reliable parameter to evaluate fetal conditions [71, 72]. Especially for antenatal assessment, STV has been reported to detect metabolic acidemia more effectively than LTV [73] and more accurately predict fetal wellbeing than umbilical artery flow velocities [74]. Furthermore, STV may be useful for providing fetal assessment during labor with fetal scalp electrodes, rather than visual interpretation [31]. Fetal autonomic systems, even with slight alterations caused by short-term MRA, may be evaluated by measuring STV, whereas a slight change in LTV cannot be detected by visual interpretation.

Nevertheless, the present study had some limitations. First, for ethical reasons, the three Rs of animal experiments, and to reduce the number of sheep used, we did not include a control group for the experiments [33, 75]. Thus, we used baseline physiological parameters as the control before MRA, and careful interpretation of the references was required. Although estimated changes in fetal parameters caused by intraamniotic inflammation itself are discussed above, the impact of inflammation on fetuses remains debatable considering the lack of comparison with a control group in a timely manner. In addition, an increase in the number of sheep treated would not have affected the results pertaining to FHR, STV, and LTV in Group 2, but the trend of an increased FHR, STV, and LTV in Group 1 following MRA administration at a dose of 200 μg/min may have been significant if a greater number of sheep were treated. Furthermore, MRA at doses of 200 μg/min and 800 μg/min was performed in the same sheep, and sub-group analyses to stratify these administrations were not performed. Second, the body weight of the maternal sheep varied, and the dosages of MRA were not adjusted accordingly. However, the dosage of MRA in this study was validated for clinical settings and presented significant effects on fetuses. Here, the ritodrine hydrochloride concentration in fetal serum increased according to the dosage and administration period of MRA, as indicated by LS-MS-MS. Because the fetal weight could not be determined during the experiment and the aim of this study was to investigate the effects of maternally administered ritodrine hydrochloride, we did not directly administer ritodrine hydrochloride to the fetuses. Third, data on intrauterine pressure and uterine blood flow were not obtained. Although intrauterine pressure is known to affect FHR variability [30], recurrent MRA would have significantly suppressed uterine contractions; in other words, pregnant sheep in the present model were not in labor during the experiment, and all sheep delivered the fetuses several days after the experiment. As MRA can reportedly reduce uterine blood flow [76, 77], this effect on FHR variability should be clarified in further studies with biophysical assessments. Finally, we did not collect the brain tissues from the fetal sheep for histopathologic analysis. Therefore, our speculation on the modified autonomic responses was based only on the physiological parameters. The excision of the fetal brain may have allowed the observation of the histopathologic changes due to intraamniotic inflammation. The role of autonomic responses during intraamniotic inflammation should be further analyzed using histopathologic findings from the brains of fetal sheep.

## Conclusions

Short-term MRA at a dose of 800 μg/min increased the FHR, STV, and LTV significantly; however, these changes were not confirmed under intraamniotic inflammation. These results implied that intraamniotic inflammation might modulate the MRA-mediated fetal autonomic responses in terms of FHR monitoring. Physicians should be aware that the effects of MRA on fetal autonomic responses may vary with the phase of inflammation; therefore, FHR monitoring should be carefully interpreted.

## Supporting information

S1 Table Absolute values for parameters investigated for each sheep. (DOCX)
