# Supplementary material for: Effects of maternal ritodrine hydrochloride administration on the heart rate of preterm fetal sheep with intraamniotic inflammation
Source: PLoS One. 2022 Mar 31;17(3):e0265872. doi: 10.1371/journal.pone.0265872 (PMC8970407; doi:10.1371/journal.pone.0265872)
Supplement: S1 Table — (DOCX) [file pone.0265872.s001.docx]

**S1 Table. Absolute values for parameters investigated for each sheep.**

| 1a.G1 MRA200 FHR | Sheep No | baseline | one h | two h |
| --- | --- | --- | --- | --- |
|  | 1 | 182.60000 | 183.90000 | 196.10000 |
|  | 2 | 203.75000 | 205.65000 | 220.00000 |
|  | 3 | 188.05000 | 197.30000 | 197.30000 |
|  | 4 | 171.60000 | 191.85000 | 215.40000 |
|  | 5 | 228.60000 | 241.35000 | 212.70000 |
| 1b.G1 MRA200 STV | Sheep No | baseline | one h | two h |
|  | 1 | 100.82860 | 76.77140 | 169.82350 |
|  | 2 | 60.37500 | 71.25640 | 147.48780 |
|  | 3 | 65.32430 | 91.23680 | 104.21050 |
|  | 4 | 61.72730 | 86.74290 | 63.02560 |
|  | 5 | 98.77780 | 79.87100 | 81.35140 |
| 1c.G1 MRA200 LTV | Sheep No | baseline | one h | two h |
|  | 1 | 55.74290 | 30.42860 | 62.14710 |
|  | 2 | 25.95000 | 26.56410 | 65.09760 |
|  | 3 | 22.05410 | 18.57890 | 30.31580 |
|  | 4 | 9.96970 | 26.71430 | 20.61540 |
|  | 5 | 54.44440 | 42.58060 | 38.72970 |
| 1d.G1 MRA200 SBP | Sheep No | baseline | one h | two h |
|  | 1 | 86.05200 | 87.45950 | 88.01300 |
|  | 2 | 65.44100 | 64.02750 | 64.27300 |
|  | 3 | 53.74200 | 53.82060 | 56.98460 |
|  | 4 | 49.86250 | 52.89850 | 52.16650 |
|  | 5 | 69.28050 | 63.51250 | 59.19750 |
| 1e.G1 MRA200 DBP | Sheep No | baseline | one h | two h |
|  | 1 | 70.36800 | 72.83900 | 73.20400 |
|  | 2 | 47.77550 | 46.49750 | 45.84850 |
|  | 3 | 37.02050 | 36.06555 | 36.21846 |
|  | 4 | 32.92900 | 33.65650 | 35.27000 |
|  | 5 | 44.39600 | 41.55850 | 36.36250 |
| 1f.G1 MRA200 FApH | Sheep No | baseline | one h | two h |
|  | 1 | 7.37900 | 7.36200 | 7.34300 |
|  | 2 | 7.36300 | 7.35100 | 7.33900 |
|  | 3 | 7.36200 | 7.39100 | 7.37500 |
|  | 4 | 7.38400 | 7.40400 | 7.39200 |
|  | 5 | 7.40500 | 7.39600 | 7.41800 |
| 1g.G1 MRA200 CO2 | Sheep No | baseline | one h | two h |
|  | 1 | 36.50000 | 39.50000 | 28.30000 |
|  | 2 | 30.90000 | 41.50000 | 36.30000 |
|  | 3 | 49.20000 | 46.60000 | 49.10000 |
|  | 4 | 42.90000 | 36.10000 | 40.20000 |
|  | 5 | 41.60000 | 41.20000 | 40.90000 |
| 1h.G1 MRA200 O2 | Sheep No | baseline | one h | two h |
|  | 1 | 28.30000 | 21.30000 | 27.20000 |
|  | 2 | 20.50000 | 21.00000 | 21.00000 |
|  | 3 | 26.40000 | 20.10000 | 20.00000 |
|  | 4 | 18.10000 | 21.20000 | 16.70000 |
|  | 5 | 24.60000 | 24.20000 | 24.60000 |
| 1i.G1 MRA200 BE | Sheep No | baseline | one h | two h |
|  | 1 | -3.20000 | -2.70000 | -9.70000 |
|  | 2 | -7.20000 | -2.40000 | -5.70000 |
|  | 3 | 2.10000 | 2.90000 | 3.00000 |
|  | 4 | 0.40000 | -1.70000 | -0.40000 |
|  | 5 | 1.30000 | 0.40000 | 1.80000 |
| 1j.G1 MRA200 lac | Sheep No | baseline | one h | two h |
|  | 1 | 1.10000 | 1.10000 | 0.90000 |
|  | 2 | 1.30000 | 1.60000 | 1.50000 |
|  | 3 | 1.70000 | 1.40000 | 1.40000 |
|  | 4 | 1.90000 | 2.20000 | 2.40000 |
|  | 5 | 1.50000 | 1.90000 | 1.90000 |
| 1k.G1 MRA200 glu | Sheep No | baseline | one h | two h |
|  | 1 | 15.00000 | 15.00000 | 14.00000 |
|  | 2 | 15.00000 | 18.00000 | 18.00000 |
|  | 3 | 17.00000 | 20.00000 | 19.00000 |
|  | 4 | 28.00000 | 30.00000 | 34.00000 |
|  | 5 | 24.00000 | 20.00000 | 29.00000 |
| 1l.G1 MRA200 rito | Sheep No | baseline | one h | two h |
|  | 1 | 0.00000 | 1.70000 | 2.40000 |
|  | 2 | 0.00000 | 4.10000 | 4.70000 |
|  | 3 | 0.00000 | 2.40000 | 2.50000 |
|  | 4 | 0.00000 | 1.90000 | 4.50000 |
|  | 5 | 0.00000 | 3.10000 | 5.00000 |
| 1m.G1 MRA200 IL6 | Sheep No | baseline | one h | two h |
|  | 1 | 0.10700 |  |  |
|  | 2 | 14.37100 |  |  |
|  | 3 | 23.81400 |  |  |
|  | 4 | 1.75300 |  |  |
|  | 5 | 13.13500 |  |  |
| 2aG1 MRA800 FHR | Sheep No | baseline | one h | two h |
|  | 1 | 147.90000 | 230.25000 | 180.65000 |
|  | 2 | 189.35000 | 238.20000 | 234.45000 |
|  | 3 | 185.60000 | 201.35000 | 222.05000 |
|  | 4 | 182.70000 | 236.30000 | 213.75000 |
|  | 5 | 184.90000 | 223.40000 | 227.90000 |
| 2b.G1 MRA800 STV | Sheep No | baseline | one h | two h |
|  | 1 | 92.44830 | 193.36840 | 130.61290 |
|  | 2 | 80.61110 | 354.70370 | 247.00000 |
|  | 3 | 60.77780 | 160.38460 | 121.18420 |
|  | 4 | 61.48570 | 92.65910 | 65.41460 |
|  | 5 | 51.05410 | 232.48720 | 134.22500 |
| 2c.G1 MRA800 LTV | Sheep No | baseline | one h | two h |
|  | 1 | 32.65520 | 122.18420 | 75.25810 |
|  | 2 | 68.66670 | 152.96300 | 122.25710 |
|  | 3 | 13.36110 | 70.15380 | 59.48720 |
|  | 4 | 7.88890 | 55.70450 | 27.43900 |
|  | 5 | 11.24320 | 97.87180 | 66.75000 |
| 2d.G1 MRA800 SBP | Sheep No | baseline | one h | two h |
|  | 1 | 82.56300 | 89.00350 | 88.08050 |
|  | 2 | 85.88600 | 92.48650 | 90.74550 |
|  | 3 | 61.90550 | 58.67750 | 59.81250 |
|  | 4 | 49.80350 | 54.37850 | 53.63450 |
|  | 5 | 58.09300 | 64.47550 | 65.20300 |
| 2e.G1 MRA800 DBP | Sheep No | baseline | one h | two h |
|  | 1 | 68.11150 | 69.25200 | 70.91650 |
|  | 2 | 73.59400 | 75.53000 | 73.82400 |
|  | 3 | 39.25750 | 36.44800 | 36.70950 |
|  | 4 | 32.56150 | 32.80200 | 33.26100 |
|  | 5 | 35.99700 | 37.03750 | 37.86000 |
| 2f.G1 MRA800 FApH | Sheep No | baseline | one h | two h |
|  | 1 | 7.33600 | 7.32800 | 7.35400 |
|  | 2 | 7.38300 | 7.40100 | 7.40800 |
|  | 3 | 7.35900 | 7.37900 | 7.37500 |
|  | 4 | 7.37600 | 7.36400 | 7.38800 |
|  | 5 | 7.38700 | 7.39600 | 7.39400 |
| 2g.G1 MRA800 CO2 | Sheep No | baseline | one h | two h |
|  | 1 | 43.40000 | 44.40000 | 43.70000 |
|  | 2 | 47.70000 | 45.70000 | 44.10000 |
|  | 3 | 49.10000 | 44.30000 | 45.90000 |
|  | 4 | 43.60000 | 40.80000 | 39.50000 |
|  | 5 | 42.40000 | 42.00000 | 42.70000 |
| 2h.G1 MRA800 O2 | Sheep No | baseline | one h | two h |
|  | 1 | 21.60000 | 24.70000 | 23.50000 |
|  | 2 | 18.10000 | 15.70000 | 16.40000 |
|  | 3 | 26.00000 | 24.80000 | 22.60000 |
|  | 4 | 19.10000 | 17.80000 | 17.70000 |
|  | 5 | 23.10000 | 20.50000 | 20.80000 |
| 2i.G1 MRA800 BE | Sheep No | baseline | one h | two h |
|  | 1 | -2.40000 | -2.50000 | -1.10000 |
|  | 2 | 3.00000 | 3.30000 | 2.90000 |
|  | 3 | 1.80000 | 0.90000 | 1.40000 |
|  | 4 | 0.20000 | -2.00000 | -1.00000 |
|  | 5 | 0.50000 | 0.90000 | 1.10000 |
| 2j.G1 MRA800 lac | Sheep No | baseline | one h | two h |
|  | 1 | 1.60000 | 2.70000 | 2.90000 |
|  | 2 | 4.40000 | 4.50000 | 5.30000 |
|  | 3 | 1.40000 | 1.80000 | 1.70000 |
|  | 4 | 2.20000 | 3.60000 | 3.70000 |
|  | 5 | 1.50000 | 2.30000 | 2.70000 |
| 2k.G1 MRA800 glu | Sheep No | baseline | one h | two h |
|  | 1 | 12.00000 | 17.00000 | 21.00000 |
|  | 2 | 18.00000 | 18.00000 | 25.00000 |
|  | 3 | 20.00000 | 31.00000 | 31.00000 |
|  | 4 | 30.00000 | 53.00000 | 63.00000 |
|  | 5 | 20.00000 | 33.00000 | 40.00000 |
| 2l.G1 MRA800 rito | Sheep No | baseline | one h | two h |
|  | 1 | 0.00000 | 4.40000 | 5.70000 |
|  | 2 | 0.00000 | 9.20000 | 10.70000 |
|  | 3 | 0.00000 | 6.50000 | 7.70000 |
|  | 4 | 0.00000 | 1.40000 | 1.60000 |
|  | 5 | 0.00000 | 1.60000 | 6.00000 |
| 2m.G1 MRA800 IL6 | Sheep No | baseline | one h | two h |
|  | 1 | 50.34500 |  |  |
|  | 2 | 77.89000 |  |  |
|  | 3 | 14.84300 |  |  |
|  | 4 | 47.36400 |  |  |
|  | 5 | 21.88400 |  |  |
| 3a.G2 MRA200 FHR | Sheep No | baseline | one h | two h |
|  | 1 | 142.60000 | 155.00000 | 155.85000 |
|  | 2 | 223.45000 | 214.90000 | 201.40000 |
|  | 3 | 198.70000 | 206.05000 | 193.40000 |
|  | 4 | 203.35000 | 205.05000 | 198.05000 |
|  | 5 | 212.65000 | 201.70000 | 195.40000 |
| 3b.G2 MRA200 STV | Sheep No | baseline | one h | two h |
|  | 1 | 72.74070 | 71.48390 | 59.56670 |
|  | 2 | 147.74290 | 233.36840 | 127.77780 |
|  | 3 | 93.44120 | 157.65000 | 111.27780 |
|  | 4 | 83.25640 | 123.23680 | 58.64860 |
|  | 5 | 103.66670 | 66.48650 | 113.64860 |
| 3c.G2 MRA200 LTV | Sheep No | baseline | one h | two h |
|  | 1 | 29.07400 | 46.51610 | 41.13330 |
|  | 2 | 75.48570 | 85.84210 | 84.83330 |
|  | 3 | 77.61760 | 118.37500 | 34.50000 |
|  | 4 | 39.84620 | 90.13160 | 31.81080 |
|  | 5 | 28.66670 | 25.83780 | 27.05410 |
| 3d.G2 MRA200 SBP | Sheep No | baseline | one h | two h |
|  | 1 | 68.55700 | 63.98900 | 59.84700 |
|  | 2 | 59.89200 | 59.50100 | 60.06450 |
|  | 3 | 48.64250 | 50.77550 | 53.75650 |
|  | 4 | 56.89400 | 57.65950 | 60.46800 |
|  | 5 | 51.40450 | 48.49750 | 52.27400 |
| 3e.G2 MRA200 DBP | Sheep No | baseline | one h | two h |
|  | 1 | 48.41150 | 46.28600 | 42.69200 |
|  | 2 | 42.80200 | 42.05800 | 41.25750 |
|  | 3 | 35.23550 | 36.52550 | 37.91050 |
|  | 4 | 35.85500 | 35.65100 | 37.83350 |
|  | 5 | 38.93700 | 34.41950 | 36.18850 |
| 3f.G2 MRA200 FApH | Sheep No | baseline | one h | two h |
|  | 1 | 7.34300 | 7.34900 | 7.33700 |
|  | 2 | 7.35100 | 7.37500 | 7.34800 |
|  | 3 | 7.39700 | 7.36800 | 7.36800 |
|  | 4 | 7.39200 | 7.42300 | 7.43400 |
|  | 5 | 7.30300 | 7.31500 | 7.33100 |
| 3g.G2 MRA200 CO2 | Sheep No | baseline | one h | two h |
|  | 1 | 47.80000 | 45.90000 | 47.40000 |
|  | 2 | 54.10000 | 50.80000 | 52.80000 |
|  | 3 | 44.30000 | 45.80000 | 46.70000 |
|  | 4 | 53.80000 | 52.80000 | 50.00000 |
|  | 5 | 54.70000 | 51.40000 | 50.70000 |
| 3h.G2 MRA200 O2 | Sheep No | baseline | one h | two h |
|  | 1 | 18.40000 | 19.90000 | 18.00000 |
|  | 2 | 23.20000 | 27.50000 | 21.30000 |
|  | 3 | 18.00000 | 18.70000 | 8.60000 |
|  | 4 | 19.60000 | 14.80000 | 16.00000 |
|  | 5 | 19.50000 | 18.50000 | 17.90000 |
| 3i.G2 MRA200 BE | Sheep No | baseline | one h | two h |
|  | 1 | -0.20000 | -0.60000 | -0.70000 |
|  | 2 | 3.80000 | 4.00000 | 2.90000 |
|  | 3 | 2.20000 | 0.80000 | 1.20000 |
|  | 4 | 6.90000 | 9.00000 | 8.30000 |
|  | 5 | 0.10000 | -0.30000 | 0.60000 |
| 3j.G2 MRA200 lac | Sheep No | baseline | one h | two h |
|  | 1 | 2.30000 | 2.80000 | 2.70000 |
|  | 2 | 1.40000 | 1.70000 | 1.90000 |
|  | 3 | 1.70000 | 2.10000 | 2.10000 |
|  | 4 | 2.10000 | 2.90000 | 2.70000 |
|  | 5 | 3.90000 | 4.90000 | 5.40000 |
| 3k.G2 MRA200 glu | Sheep No | baseline | one h | two h |
|  | 1 | 14.00000 | 21.00000 | 22.00000 |
|  | 2 | 14.00000 | 22.00000 | 28.00000 |
|  | 3 | 21.00000 | 44.00000 | 40.00000 |
|  | 4 | 7.00000 | 16.00000 | 19.00000 |
|  | 5 | 16.00000 | 19.00000 | 23.00000 |
| 3l.G2 MRA200 rito | Sheep No | baseline | one h | two h |
|  | 1 | 0.00000 | 4.60000 | 4.80000 |
|  | 2 | 0.00000 | 1.30000 | 2.00000 |
|  | 3 | 0.10000 | 2.30000 | 2.60000 |
|  | 4 | 0.00000 | 1.90000 | 3.50000 |
|  | 5 | 0.00000 | 1.00000 | 2.10000 |
| 3m.G2 MRA200 IL6 | Sheep No | baseline | one h | two h |
|  | 1 | 195.96200 |  |  |
|  | 2 | 125.10200 |  |  |
|  | 3 | 695.62600 |  |  |
|  | 4 | 544.15700 |  |  |
|  | 5 | 146.71300 |  |  |
| 4a.G2 MRA800 FHR | Sheep No | baseline | one h | two h |
|  | 1 | 192.35000 | 190.95000 | 187.75000 |
|  | 2 | 185.80000 | 188.90000 | 192.55000 |
|  | 3 | 200.30000 | 196.35000 | 197.00000 |
|  | 4 | 182.85000 | 180.45000 | 180.80000 |
|  | 5 | 187.05000 | 187.50000 | 195.30000 |
| 4b.G2 MRA800 STV | Sheep No | baseline | one h | two h |
|  | 1 | 103.41670 | 122.62160 | 131.65710 |
|  | 2 | 117.37140 | 342.62160 | 138.27030 |
|  | 3 | 77.10260 | 115.63160 | 85.78380 |
|  | 4 | 99.34290 | 51.02940 | 51.71430 |
|  | 5 | 92.66670 | 120.36110 | 153.47500 |
| 4c.G2 MRA800 LTV | Sheep No | baseline | one h | two h |
|  | 1 | 46.52780 | 86.75680 | 95.45710 |
|  | 2 | 75.14290 | 166.83780 | 72.43240 |
|  | 3 | 50.25640 | 89.15790 | 42.21620 |
|  | 4 | 21.11430 | 29.82350 | 28.62860 |
|  | 5 | 35.86110 | 35.41670 | 43.55000 |
| 4d.G2 MRA800 SBP | Sheep No | baseline | one h | two h |
|  | 1 | 56.36444 | 52.42950 | 47.47950 |
|  | 2 | 54.03700 | 52.73350 | 50.91050 |
|  | 3 | 50.40650 | 50.26550 | 51.21650 |
|  | 4 | 52.61200 | 52.20650 | 52.45500 |
|  | 5 | 50.51200 | 49.16950 | 51.16000 |
| 4e.G2 MRA800 DBP | Sheep No | baseline | one h | two h |
|  | 1 | 41.37000 | 38.77700 | 36.70450 |
|  | 2 | 38.56850 | 35.07150 | 33.93650 |
|  | 3 | 36.19550 | 35.56350 | 37.03850 |
|  | 4 | 36.01300 | 35.07800 | 34.38800 |
|  | 5 | 37.53750 | 35.74150 | 34.31550 |
| 4f.G2 MRA800 FApH | Sheep No | baseline | one h | two h |
|  | 1 | 7.34200 | 7.31300 | 7.30500 |
|  | 2 | 7.35400 | 7.34800 | 7.32300 |
|  | 3 | 7.38300 | 7.33800 | 7.34600 |
|  | 4 | 7.45700 | 7.42500 | 7.43600 |
|  | 5 | 7.40700 | 7.38100 | 7.40200 |
| 4g.G2 MRA800 CO2 | Sheep No | baseline | one h | two h |
|  | 1 | 50.10000 | 50.70000 | 49.00000 |
|  | 2 | 49.20000 | 50.80000 | 51.40000 |
|  | 3 | 44.90000 | 47.60000 | 44.50000 |
|  | 4 | 44.70000 | 43.60000 | 43.30000 |
|  | 5 | 46.10000 | 49.50000 | 46.40000 |
| 4h.G2 MRA800 O2 | Sheep No | baseline | one h | two h |
|  | 1 | 16.00000 | 16.00000 | 15.20000 |
|  | 2 | 23.60000 | 19.50000 | 18.50000 |
|  | 3 | 15.60000 | 16.30000 | 16.40000 |
|  | 4 | 24.50000 | 17.10000 | 16.30000 |
|  | 5 | 16.00000 | 15.50000 | 19.70000 |
| 4i.G2 MRA800 BE | Sheep No | baseline | one h | two h |
|  | 1 | 0.80000 | -1.70000 | -2.20000 |
|  | 2 | 1.70000 | 2.00000 | 0.50000 |
|  | 3 | 1.40000 | -0.40000 | -1.30000 |
|  | 4 | 7.00000 | 3.90000 | 4.60000 |
|  | 5 | 3.90000 | 3.60000 | 3.70000 |
| 4j.G2 MRA800 lac | Sheep No | baseline | one h | two h |
|  | 1 | 2.60000 | 3.50000 | 4.30000 |
|  | 2 | 1.40000 | 2.10000 | 2.70000 |
|  | 3 | 2.10000 | 3.50000 | 3.50000 |
|  | 4 | 1.60000 | 2.10000 | 2.50000 |
|  | 5 | 2.80000 | 3.70000 | 3.80000 |
| 4k.G2 MRA800 glu | Sheep No | baseline | one h | two h |
|  | 1 | 13.00000 | 26.00000 | 31.00000 |
|  | 2 | 14.00000 | 24.00000 | 32.00000 |
|  | 3 | 11.00000 | 20.00000 | 21.00000 |
|  | 4 | 12.00000 | 12.00000 | 16.00000 |
|  | 5 | 11.00000 | 13.00000 | 22.00000 |
| 4l.G2 MRA800 rito | Sheep No | baseline | one h | two h |
|  | 1 | 0.00000 | 2.50000 | 2.60000 |
|  | 2 | 0.00000 | 2.50000 | 4.10000 |
|  | 3 | 0.10000 | 4.30000 | 4.90000 |
|  | 4 | 0.00000 | 5.60000 | 10.10000 |
|  | 5 | 0.00000 | 10.50000 | 26.20000 |
| 4m.G2 MRA800 IL6 | Sheep No | baseline | one h | two h |
|  | 1 | 387.81500 |  |  |
|  | 2 | 971.77500 |  |  |
|  | 3 | 976.06500 |  |  |
|  | 4 | 148.93500 |  |  |
|  | 5 | 482.34100 |  |  |
